# Task-shifting for point-of-care cervical cancer prevention in low- and middle-income countries: a case study from Uganda

**DOI:** 10.3389/fpubh.2023.1105559

**Published:** 2023-07-28

**Authors:** Judith Auma, Allan Ndawula, James Ackers-Johnson, Claire Horder, Maaike Seekles, Veena Kaul, Louise Ackers

**Affiliations:** ^1^Hampshire Hospitals NHS Foundation Trust, Basingstoke, United Kingdom; ^2^Kataraka Health Centre, Knowledge for Change (K4C), Fort Portal, Uganda; ^3^Knowledge for Change, University of Salford, Salford, United Kingdom; ^4^School of Health and Society, University of Salford, Salford, United Kingdom; ^5^Liverpool School of Tropical Medicine, Liverpool, United Kingdom; ^6^Mid Yorkshire Hospitals NHS Trust, Wakefield, United Kingdom

**Keywords:** cervical cancer, prevention, task-shifting, frugal innovation, geographic information systems, results based finance

## Abstract

Cervical cancer remains the leading cause of female cancer deaths in sub-Saharan Africa. This is despite cervical cancer being both preventable and curable if detected early and treated adequately. This paper reports on a series of action-research ‘cycles’ designed to progressively integrate a comprehensive, task-shifted, point-of-care, prevention program in a community-based public health facility in Uganda. The work has been undertaken through a UK-Ugandan Health Partnership coordinated by Knowledge for Change, a UK-registered Charity. The intervention demonstrates the effectiveness of task-shifting responsibility to Community Health Workers combined with the use of Geographic Information Systems to strategically guide health awareness-raising and the deployment of medical devices supporting respectful and sustainable point-of-care screen-and-treat services. The integration of this with public human immunodeficiency virus services demonstrates the ability to engage hard-to-reach ‘key populations’ at greatest risk of cervical cancer. The findings also demonstrate the impact of external influences including the Results Based Financing approach, adopted by many foreign Non-Governmental Organizations. The model presents opportunities for policy transfer to other areas of health promotion and prevention with important lessons for international Health partnership engagement. The paper concludes by outlining plans for a subsequent action-research cycle embracing and evaluating the potential of Artificial Intelligence to enhance service efficacy.

## 1. Introduction. Cervical cancer: a case of neglect

In Uganda, cervical cancer is the most common cause of both cancer-related incidence (54.8 per 100,000) and cancer-related deaths (40.5 per 100,000) (1). Eighty percent of patients present late with advanced stage (often terminal) disease (2). Late patient presentations are attributed to low levels of knowledge among health care providers and the public about cervical cancer and prevention strategies and minimal access to available (and free) public screening services (2, 3). The 2010 National Strategic Plan for Cervical Cancer Prevention and Control prioritized 3 areas (3). The first 2 of these include an emphasis on health education and awareness-raising:

1. Human Papillomavirus (HPV) vaccination of 10–14-year-old girls
2. Low-cost screening using Visual Inspection with Acetic Acid (VIA)
3. Treatment of early dysplasia (cervical intraepithelial neoplasia) using cryotherapy

While HPV vaccination has become the primary preventive intervention in high income settings, Uganda’s HPV vaccination program, targeting girls aged 10–14 in primary schools, has achieved only about 20% uptake (4). Progress has been substantially impacted by the immediate and long-term effects of extended school closures during the COVID-19 pandemic and subsequent Ebola outbreaks. For the foreseeable future, this implies continued emphasis on screen-and-treat programs working in parallel with HPV vaccination. A key goal of Priorities 2 and 3 was to have 80% of eligible women screened and treated for precancerous lesions (3). Despite these intentions, Uganda’s national screening program faces considerable implementation gaps. In practice, screening in Uganda is erratic and absent in many regions (5). Unfortunately, there is very limited (or no) public funding for such programs which suffer from acute donor-dependency (6, 7). Uptake of screening services that do exist are negatively impacted by limited access to facilities, compounded by the costs associated with services, travel, and wait times (8, 9). There is also a shortage of trained screening providers (10). This explains the low lifetime screening rate of between 4.8 and 30% (11), and the continued prevalence of advanced disease and mortality (2).

Cervical screening guidelines in Uganda are based on a ‘see-and-treat’ approach targeting women aged 25 to 49 years. VIA is the main screening procedure used. In theory, women diagnosed with positive mild–moderate precancerous lesions (diagnosed through VIA) should then be treated, in the community, using cryotherapy. In practice, even Non-Governmental Organizations (NGOs) that constitute the private ‘not-for-profit’, sector levy significant charges for such treatment. National Guidelines (until recently) stipulated that women who test positive for Human Immunodeficiency Virus (HIV) should be screened annually while HIV-negative women should undergo cervical screening every 3 years. Midwives and nurses are the primary providers of cervical cancer screening as well as treatment (3).

Knowledge for Change (K4C) is a UK and Ugandan registered NGO focused on health systems change.^1^ At the time of the commencement of the Knowledge for Change (K4C) screen-and-treat service in Fort Portal (Uganda), the city had no public facility offering free cervical screening services. This paper presents a Community Case Study documenting a complex intervention (defined through a series of action-research cycles) drawing out the potential for scale-out of see-and-treat cervical cancer prevention in Low- and Middle-Income Countries (LMICs).

## 2. The K4C screen and treat intervention and action-research methodology

It is customary in research papers to outline the specific methods used for a time-limited study with a clearly specified objective. The overall long-term objective in this work was to address the lack of preventive cervical cancer screening services and, in so doing, find ways to sustainably reduce the mortality associated with this preventable disease. In practice, K4C has learnt that health systems change is rarely a paradigm-shifting process but happens through closely contextualized and carefully evaluated progressive incremental change (6). External influences beyond the control of individual projects in Aid-driven health systems have considerable potential to disrupt and even undermine planned interventions. The authors share complex positionalities as health workers, charity actors and trustees, volunteers, and academic researchers. Our approach to research is best described as action or implementation research. We have described the challenging and unpredictable nature of this approach elsewhere (6) and alluded to the necessity of process continuity. Action-research does not have simple start and end-dates and objectives inevitably need to change over time in response to changing contexts and opportunities. The publication of results in this paper marks a stage in an unpredictable journey, characterized by a series of action-research cycles each bringing new knowledge and expertise to the wider intervention. The intervention commenced some years ago with measures to promote respectful care in service delivery at Kagote Health Centre, Uganda (12). Respectful care provides the essential foundation for any intervention to improve access to public services. The decision to develop a preventive clinic in Kagote in 2017 kick-started a sequence of evolving action-research cycles starting with device procurement then moving onto staff training, public awareness-raising and service integration with HIV clinics. As staff trained under our program were routinely rotated by the District Health Office into other facilities, screen-and-treat services were extended to these public health centres. In practice, this took place in a more sporadic fashion and in the absence of a systematic health awareness intervention. The paper documents the development of K4C’s Model for community-based cervical screening with interventions and the results associated with them reported sequentially.

## 3. The action-research cycles

Section 3 documents a series of 4 action-research cycles. They are distinguished here to allow discussion of the specific programmatic elements they concern (device procurement, capability-enhancement, the use of Geographic Information Systems to guide awareness-raising and service integration with HIV care). The distinction also maps the chronology of intervention. In reality, action-research is necessarily a ‘messy’ process (13, 14) and cycles overlap and interface with each other.

### 3.1. Cycle 1: point-of-care device procurement

Cycle 1 focused on the selection of appropriate devices to support sustainable task-shifting in Community-based facilities. In 2017, K4C established the first screen-and-treat cervical cancer prevention service in a Ugandan community-based public health facility (Kagote Health Centre). K4C grew increasingly concerned at the ‘outreach’ or ‘health camp’ model to partnership working. This approach, delivering services at remote locations and often through organizations operating in parallel to public services, has been explicitly fostered by the private (ostensibly) ‘not-for-profit’ sector. This approach is immediately attractive to foreign ‘donors’ driven by numerical ‘outcome’ measures and to hosting organizations that operate a ‘vending-machine’ approach to income generation. K4C had found itself caught up in this approach as local organizations encouraged us to fund screening out-reaches. We soon stopped this activity as we were concerned about the ethics of offering free screening to women in the absence of free preventive treatment of eligible precancerous lesions. The first action-research cycle then focused on the procurement of a suitable point-of-care device. Biomedical engineering expertise lies at the heart of K4C’s work and we were very aware of the risks associated with the procurement of equipment (such as the popularly used cryotherapy devices) reliant on on-going supplies of consumables or reagents. In practice, much of this equipment lies unused in health facilities waiting for donor funding for consumables (15). With advice from its specialist biomedical engineer and informed by recent research on the relative merits of thermocoagulation – also known as ‘cold coagulation’ or thermal ablation (16, 17), K4C procured a rechargeable thermocoagulation device. This device is hand-held, rechargeable and no bigger than a standard hairdryer.

Existing screening programmes in Uganda were using the naked-eye approach. Visual Inspection with Acetic Acid (VIA) is generally used in public and not-for-profit sectors in Sub-Saharan Africa where PAP Smear tests or testing for the presence of Human Papillomavirus (HPV)^2^ is regarded by national authorities and the World Health Organization to be inefficient (20–22). The VIA approach has been effectively task-shifted to midwives and nurses in Uganda, many of whom will have received varying levels of training (again almost exclusively by NGOs). Concerned about the efficacy of this random approach to training, K4C embarked on a comprehensive training program including training in the use of a new device, developed by a company in Israel to enhance and quality assure the VIA process. The Enhanced Visual Assessment (EVA) device is effectively a colposcope mounted on a Samsung phone that captures a high-quality image of the cervix and uploads that, along with key patient data, onto a secure database.^3^ The password-protected database is then accessible in real time from anywhere in the world. This rapid point-of-care diagnostic technology gives a better view of the cervix and provides opportunities for remote review and audit of clinical decision-making. Critically, in the K4C Model, it has supported capacity-building through a virtual volunteering (or telemedicine) approach. Beery (23) co-founder of Mobile ODT, the company manufacturing the EVA device, describes the role that it can play in supporting training and quality assurance mechanisms in cervical screening programs in developing countries. This approach connects to a fast-emerging knowledge base around digital health more generally and point of care diagnostics particularly in resource-poor and geographically remote environments (24). It also responds to concerns about potential ‘diagnostic drift’ associated with one-time, poorly integrated, fly-in fly-out, NGO-led training. The limited impact of such approaches on health worker behavior have been reported in areas such as neo-natal resuscitation training and emergency obstetric care (6, 25). A systematic review of interventions to improve health worker performance in LMICs found that ‘one time training interventions result in very low to no learning outcomes (26).

### 3.2. Cycle 2: capability-enhancement for effective task-shifting

The introduction of new devices and services demands attention to staff capability and training. Cycle 2 focused specifically on knowledge transfer and training. Task-shifting is defined by the World Health Organization (WHO) as ‘the rational re-distribution of tasks (…) from highly qualified health workers to health workers who have fewer qualifications in order to make more efficient use of the available human resource’ (27). We have argued elsewhere (25) that the concept of task-shifting is often best characterised as task-dumping (dumping tasks on less well-paid staff, without the necessary training and often falling out with their role specification exposing them to risk of professional malpractice). To avoid the risks associated with delegating tasks to staff who were not trained in their initial education or through continuing professional development in such tasks, the planned program required an initial phase of health worker training. The multi-disciplinary knowledge mobilization team included K4C volunteers (midwives, nurses and doctors), Ugandan midwives and a doctor employed directly by K4C, and the midwives, nurses and Village Health Workers employed at the local health facilities. The training included 2 two-week blocks of intensive on-site training (of a total of 40 health workers) followed by long term, continuous, mentoring supported by the EVA-device’s database function. In practice, this involved fortnightly reporting of cases to the UK experts which could be checked against the database. As expertise developed in-country, the UK team now responds only to cases referred to them.

Evaluation of the initial training (using pre- and post-training tests) demonstrated familiarity with the VIA technique. However, as the EVA system was new, further training was required on data input, how to take high quality colposcopic photographs and interpret them in real time. Overall, the trainees were quick to learn how to use the EVA, and the images provided opportunities for patient education, peer-peer learning, targeted treatment and later, clinician to clinician support. We soon learnt that screening required two healthcare professionals when using the EVA (one to perform the procedure and one to take the images) as it was difficult to take high quality images while maintaining infection prevention control measures and keeping the cervix in view. This had the advantage though of implementing K4C’s principle of co-working and co-presence (6) ensuring the trainee received peer support and a second opinion while learning to use the EVA. Interviews with health workers reported high satisfaction among patients who were pleased to receive immediate feedback on their examination using the images, which in turn helped the trainees to explain the findings and offer treatment. We found that women were very interested in the images and did not feel embarrassed by the strategy. In Ghana, nurses training in VIA using a smartphone colposcope and their patients had a similar positive experience using this approach (28). Training extended to the use of the thermocoagulation device, as very few Ugandan health workers in public settings had received training in treatment methods. The WHO recommends that trained nurses and midwives perform thermal ablation in addition to physicians (17). For women who were VIA positive, with lesions eligible for ablative treatment (according to WHO guidelines), it proved to be easy for the midwives to use and acceptable to the patients. As there was a low VIA positive rate during training, it was not possible for all trainees to practice but they were later supported by their colleagues who had had the opportunity and together confidence was gained in the thermal ablation technique. The Commonwealth Professional Fellowship Scheme enabled further enrichment through the training of 6 colleagues in the UK^4^.

**Figure 1:**
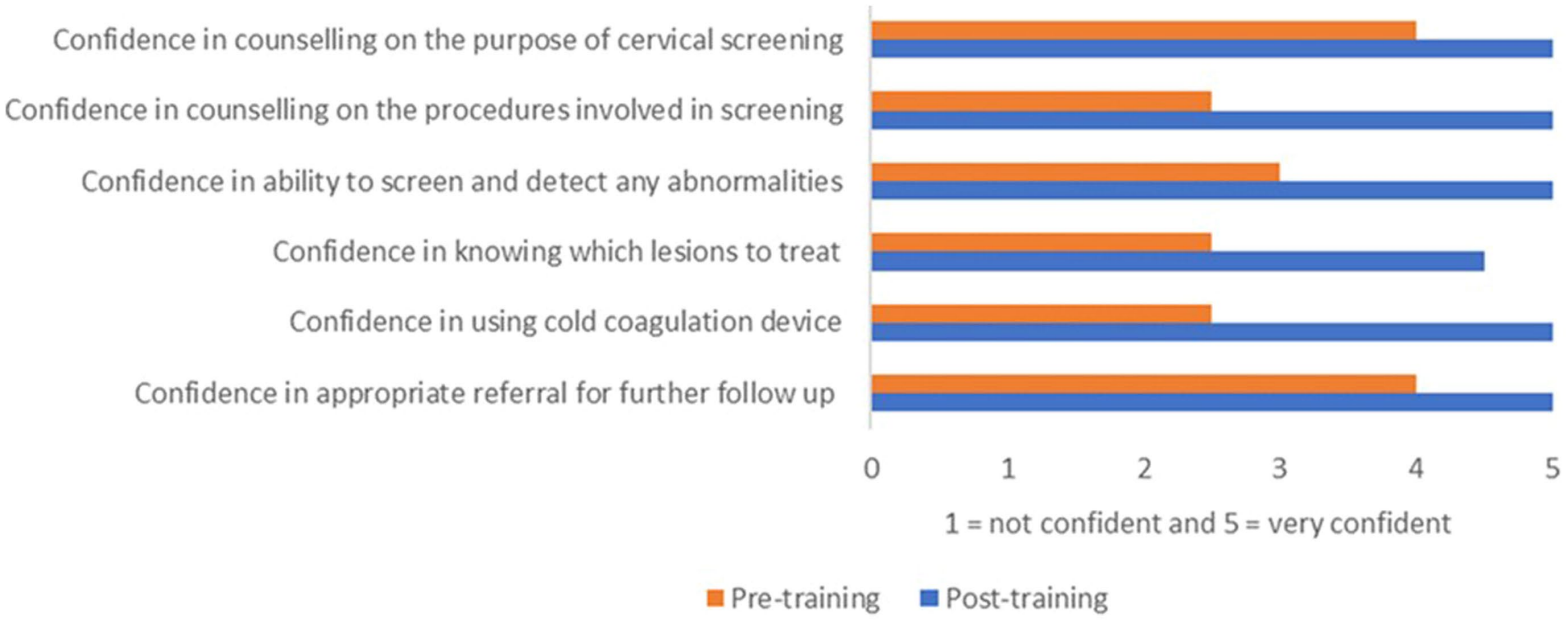
Confidence of the healthcare providers pre- and post-training (K4C).

Fit-for-Purpose, contextualized, training materials were developed which gave a comprehensive overview of the basic histology of the cervix, through to cervical screening methods, investigation and management of cervical cancer; these have provided a continuous resource to follow-up training.^5^ The course included opportunities for midwives to practice counseling and consenting women for screening with feedback from trainers. It also included simulating thermal ablation using models of the cervix made from shoeboxes and raw meat. Prior to training, participants were asked to complete a multiple-choice questionnaire assessing their knowledge of local screening eligibility criteria, cervical anatomy and types, identification of benign and precancerous cervical lesions and screen and treat methods using VIA. Generally, at baseline, the healthcare providers’ knowledge about cervical screening was fairly good. The majority correctly identified HPV as the causal agent and acknowledged the significance of cervical screening toward early detection and prevention of cervical cancer. They also had good knowledge of the screening target age group and the screening intervals. However, most participants found it challenging to identify the transformation zone of the cervix, distinguish benign lesions from abnormal changes, and deciding when to treat. The majority also did not know the appropriate time to view the cervix for any changes after application of acetic acid. This raised a significant area to focus on while conducting training and/or refresher courses. The post-test assessment questionnaire showed significant improvement in overall knowledge of cervical screening using VIA and treatment of minor lesions. This is congruent with previous studies identifying training as an effective intervention in cervical cancer prevention (29, 30). Participants’ confidence was also assessed on a 5-point Likert scale; Figure 1 illustrates overall improvements in confidence. Slightly lower confidence was seen in understanding which lesions to treat compared to the other components. Previous studies have reported the subjective nature of VIA which makes it challenging to accurately identify abnormal changes (31). This is because the demarcated acetowhite staining on VIA positive women can be suggestive of HPV infection, inflammation, metaplasia, or dysplastic changes (32, 33). This underlines the need for continual experiential practice, regular refresher courses and mentoring to improve confidence and maintain necessary skill sets.

K4C recognizes the limitations of formal one-off training interventions and always combines these with continuous mentoring and support. This has taken the form of co-working (where volunteers and K4C staff work in co-present relationships with local staff) and telemedicine support. The EVA device is uniquely positioned as images can be shared in real time through the system’s encrypted online image-sharing database, supporting on-going discussion over diagnoses. A fortnightly case reporting system has also enabled the team to flag cases of interest or concern through this virtual mentoring process. As expertise and confidence has developed, we have found that more cases can be resolved through in-country discussion. It is important to note that K4C had been actively involved in the development of ‘respectful care’ in Kagote Health Centre and this laid the essential foundations of trust and mutual respect (34).

Once the screening facility was established and staff positioned to provide services, we were initially (naively) disappointed at the uptake. Support from the UK’s Small Charities Challenge Fund (SCCF)^6^ provided the opportunity to develop an ambitious awareness-raising program.

### 3.3. Cycle 3: systematizing health awareness raising using geographic information systems (GIS)

Cycle 3 responded to the need to raise awareness among eligible women about cervical cancer, the importance of preventive action and the screen-and-treat process. The WHO identifies community awareness-raising and health education as critical components of preventive screening to ensure optimal geographical coverage, treatment adherence and challenge common taboos (35). To achieve this, the Ugandan Ministry of Health proposed a target of 90% of women aged 15–49 years to be reached through Information, Education, and Communication (IEC) materials about cervical cancer (MOH, 2010). Under this system, Uganda’s Village Health Teams (VHTs) are the primary vehicle for public health engagement primarily through outreach activities. Nominally, VHTs are unpaid volunteers embedded in local communities. In practice, these ‘volunteers’ are typically remunerated by Ministry and NGO actors on specific missions (including childhood immunization, family planning and HIV awareness).

Concerned to optimize demand for the new screening service, K4C took the decision to mobilize the local VHTs working alongside midwives and nurses in an ambitious community outreach program. As noted above, this included an initial phase of multi-disciplinary training to ensure that VHTs understood cervical cancer and the role that early prevention can play and anticipate some of the barriers to screening among local women.

The interviews with health workers conducted as part of the evaluation emphasize both the importance of using VHTs (given their rapport with local communities) and more informal relationships but also the importance of ensuring that health education messages are evidence-based:

The community health workers/VHTs (…) could be a better option cause they are familiar with the clients and the clients are okay with them. If they are the ones to pass on the message to the clients, then there would be no cause for alarm (HCP 7 - Records personnel).

Most times I say cooperation is good. The VHTs have been very helpful. If they are seated [with the other health workers] and the VHTs give the talk to the women, it is very good. Then if the health worker also tops up the voice? I think this thing would sound more beautiful than if only left to the VHT. Others may think the VHTs don’t have much information, but if they cooperate I think the voice would be heard so good (HCP 5 – Midwife).

We were also aware that VHT mobilizations are typically quite random. Although they are selected to represent their local communities as a rule health education programs, whether organized through the government or by NGOS, typically provide limited direction (or monitoring) of geographical coverage. In early trials we found that VHTs tended to focus, for example, on the very local market area. Perhaps because of the volume of people present but also for pragmatic reasons; less far to walk and convenient to combine with shopping. We wanted the intervention to take a more strategic approach to public awareness-raising supported by fine grained, geo-health data. The decision was taken to pilot the use of Geographical Information Systems (GIS) to guide outreach work combining health education with a community survey. The GIS approach was informed by a model used in rural Nigeria to determine the prevalence of hypertension and its comorbidities leading to mortality (36).

The community survey was designed to function on a tablet device using Epicollect, an Open Data Source capable of capturing location data (37). The survey acted as a census to determine women’s previous cervical screening experiences, capture key demographic characteristics and inform our approach to cervical cancer awareness. It was conducted by trained VHT/midwife pairs. Once the team established the presence of an eligible respondent (a woman aged 18–60) a short interview was conducted, and the responses saved on a tablet or mobile phone. The survey was followed by a short health education talk on the process and effectiveness of cervical screening and guidance on how to access the free public service. Women were also provided with an information leaflet translated into local languages. The community survey and mapping activities were planned to take place twice-weekly, prior to the twice-weekly clinics to reduce any delays and facilitate immediate responses. In practice, the awareness raising was so successful in terms of generating demand, we had to tailor the frequency of the outreach work to ensure that we could deliver a high-quality service and minimize waiting times which we knew were a deterrent to women. The outreach intervention involved 48 days (usually 2 days/week prior to clinics) over a 6-month period from April 2019. A total of 2014 participants were interviewed during the door-to-door visits.^7^ The average age of participants was 30 years, ranging from 18 to 86 years.

#### 3.3.1. Survey and GIS mapping results

**Figure 2:**
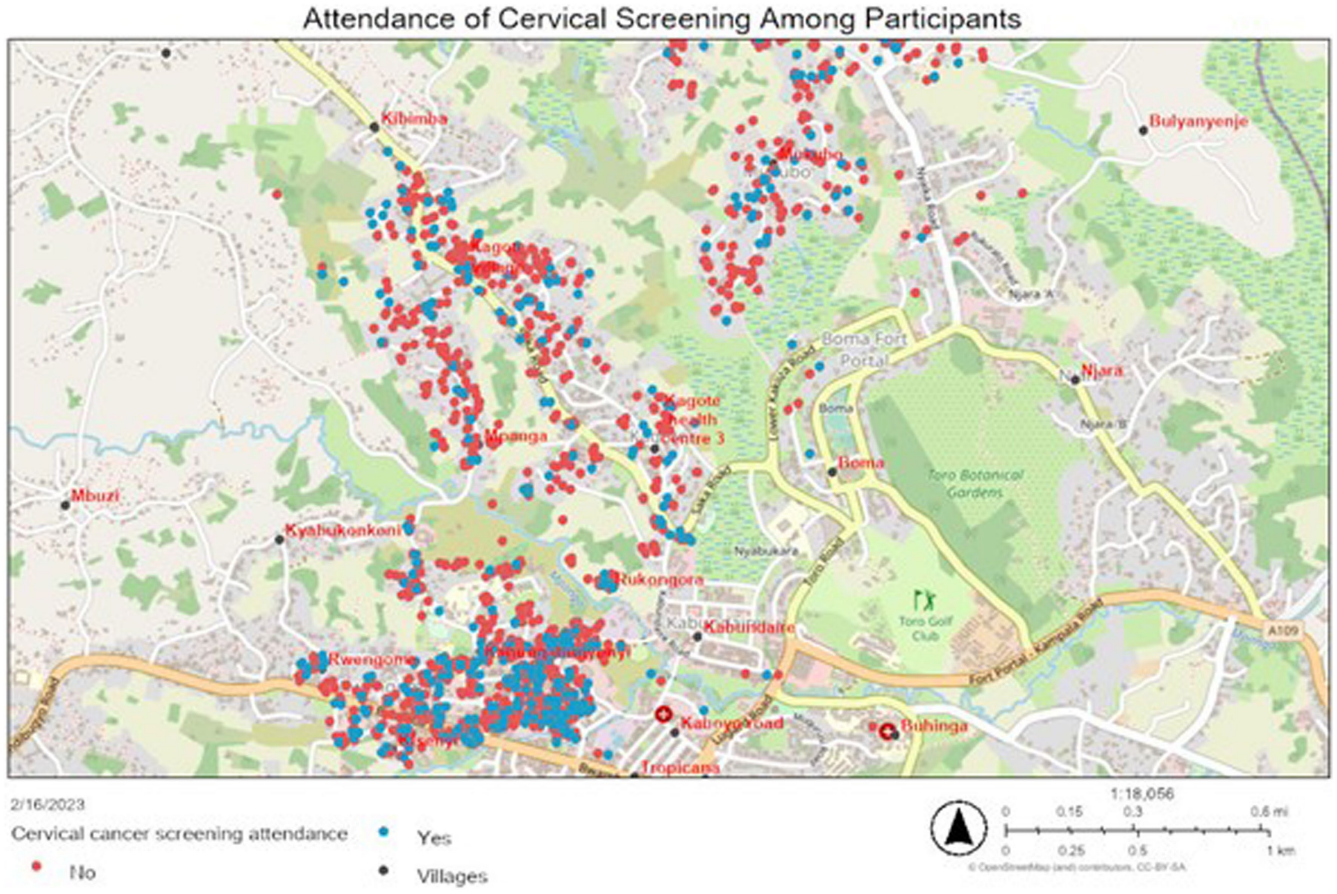
Geographical distribution of women by previous screening attendance (37).

The GIS approach supported a dynamic analysis integrating location data with a range of other data collected from the survey to produce interactive visual maps describing participants’ experiences in association with their location. By way of example, Figure 2 shows the distribution of participants that reported having previously attended cervical screening:

It is surprising that participants who attended screening (the blue dots) lived in the same communities as those who never attended, suggesting either limited peer-to-peer influence or perhaps that they have gained awareness but do not feel the need to access screening at this point. The problem with this in the case of cervical cancer is that this is a largely symptom-free condition until the cancer is advanced. This raises the need to further explore (and challenge) factors leading to low uptake such as cultural myths and beliefs, costs of screening, and lack of time (11, 38). In addition to this substantive data (on screening experience), Figure 2 demonstrates the power of the GIS approach in terms of strategically guiding health awareness work. The area most densely populated with cases (to the south of Kagote Health Centre) is the area traditionally frequented by VHTS in outreach work. Continuous mapping of results enabled the project manager to guide, and, where necessary, provide transport to extend the geography of the intervention. The approach could also be scaled-out to support the planning of future cervical cancer prevention and control interventions. In practice, we have seen patient journeys increase as the reputation and awareness of the service has grown over time; this includes cases of women traveling from outside the health district to access services at Kagote. Women have a right, under MOH protocols, to access any public service and the widening catchment area can be seen as an indicator of service availability and acceptability. The necessity of travel does raise issues about the need to extend the service to other, more distributed, community health centres.

Respondents who said they had previously been screened were asked to name the facility they accessed. The purpose of this question was to identify the kinds of facilities women had been attending. As noted above the survey took place a short time after screening was introduced at Kagote. Of the 399 respondents who had previously attended screening the largest group had attended public facilities with 131 (33%) identifying public hospitals. A total of 81 cases (20.3%) involved screening in community health facilities as envisaged in the MOH Strategy. This latter group is dominated by Kagote residents (61 of the 81 cases). A further 38.6% named private facilities (including both for and not-for-profit centres). Most of this group (100 out of 154) named key NGO providers (Reproductive Health Uganda, Marie Stopes and Mildmay). An additional 31 women said they had been screened in outreach camps in the past.

#### 3.3.2. Sources of information on cervical cancer and screening

One of the questions the survey sought to assess concerned the sources of information women relied upon when making decisions about reproductive health and/or cancer screening. Participants were presented with a range of options and invited to identify which they relied upon for health information. Table 1 confirms the findings of Uganda’s National Media Access Statistics that confirm reliance on radio as the main source of public information rated at 65% (39). This finding encouraged us to use local radio stations to inform women about the need for and access to free cervical screening.

**Table 1:** Main sources of health education by age group.

Age group	Radio % (*n*)	Word-of-mouth % (*n*)	VHTs % (*n*)	TV % (*n*)	Newspaper % (*n*)	Other (%)
18–24 (*n* = 571)	62.2 (355)	35.0 (200)	25.4 (145)	26.6 (152)	4.0 (23)	14.4 (82)
25–34 (*n* = 918)	60.5 (555)	40.0 (367)	30.9 (284)	23.4 (215)	3.4 (31)	20.5 (188)
35–44 (*n* = 353)	58.6 (207)	36.8 (130)	31.7 (112)	27.2 (96)	5.7 (20)	20.1 (71)
45+ (*n* = 172)	75.0 (129)	34.3 (59)	29.7 (51)	33.1 (57)	7.6 (13)	11.0 (19)
Total	61.9 (1246)	37.5 (756)	29.4 (592)	25.8 (520)	4.3 (87)	17.9 (360)

After word of mouth, Village Health Workers formed the next most cited source of health information supporting our decision to actively train and deploy these cadres. The survey did not specifically ask about social media and we would anticipate this becoming a significant source of information and misinformation in the future. Table 1 shows that, for now, even younger age groups are very reliant on radio and VHTs.

The survey also asked whether respondents had attended cervical screening before. Table 2 summarizes the results cross tabulated by age group, educational level and information source.

**Table 2:** Previous screening attendance by age group, education level and information source.

	Reported previous screening % (*n*)
Total (*n* = 2014)	20.0 (402)
Total 25+ (*n* = 1443)	24.2 (349)
*Age group*	
18–24	9.3 (53)
25–34	20.4 (187)
35–44	30.3 (107)
45+	32.0 (55)
*Education level completed*	
None	22.1 (25)
Primary	19.9 (108)
Secondary	18.4 (167)
University	22.6 (102)
*Information source*	
TV	24.0 (125)
VHT	23.6 (140)
Newspaper	23.0 (20)
Radio	19.9 (248)
Word-of-mouth	18.1 (137)
Other	19.4 (70)

Prior to the health education program, only 20% (402) respondents had attended cervical screening at the time of the survey. Some significant differences in percentages screened across age groups, with (perhaps inevitably) those in the youngest age group (18–24) least likely to have received cervical screening. No significant differences were seen in the likelihood of screening by the educational levels attained. Interestingly, for those women who have received screening in the past the sources of information were much more balanced.

### 3.4. Cycle 4: integrating cervical screening and HIV clinics

Cycle 4 responded to the specific risks that women who are HIV positive are exposed to and the opportunities to capture those women accessing regular Anti-retroviral medications. Action-Research cycle 4 extended the previous literature review to focus specifically on the relationship between cervical cancer and HIV prevalence. A previous study evaluating cervical screening techniques in Uganda, found that HIV positive women had a higher prevalence of precancerous lesions than HIV negative women; 12.9% vs. 1.7%, respectively (40). HIV positive women have also been shown to have a higher rate of persistent multiple high-risk HPV infections (41) and higher incidence of both precancerous and invasive cervical cancer lesions (42). Cobucci et al. (43) attributes this to the long-term effects of increased access to antiretroviral therapy that has lengthened life expectancy for HIV positive women, and thus exposing them to the risk of developing other AIDS-defining cancers. HIV also weakens the natural cell-mediated immune responses that are required to clear HPV infection increasing the likelihood of an HIV positive woman’s cervical cells developing into premalignant lesions and advancing to invasive cancer (44–46). UNAIDS/WHO (17) estimates that about 17.3 million women form almost half of the total number of HIV positive individuals worldwide and of these, 13.2 live in sub-Saharan Africa. Uganda is only second to South Africa where 2,363 individuals get infected with HIV every week. Currently in Uganda, as regards to UNAIDS (47) data, 1.4 million people were HIV positive in 2022, and about 17 000 AIDS-related deaths were reported, with an estimated HIV prevalence among adults (aged 15–49) standing at 5.1%. Women are more affected by HIV than men, with 6.5% of adult women aged 15–49 being HIV positive compared to 3.6% of men. Additionally, HIV prevalence is almost four times higher among females aged 15 to 24 than males of the same age (48). Sia et al. (49) attribute this gender inequality in HIV/AIDS prevalence in Uganda to the gender differences in the distributions of observable HIV/AIDS risk factors (i.e., sociodemographic characteristics, sexual behaviors, and HIV/AIDS awareness) between women and men. For instance, the lower socioeconomic status of women predisposes them to transactional and intergenerational unprotected sexual relations that may increase their vulnerability to HIV (50–53). Additionally, poorer and less-educated women may lack the knowledge needed to adopt HIV risk-reducing behavior (54).

We have noted the importance of respectful care in ensuring optimal access for these ‘hard-to-reach’ patients. As the screening work was developing one of K4C’s Ugandan doctors identified the lack of integration between the HIV-clinic at the local health centre and the cervical screening clinic (about 10 meters apart). This stimulated a further action-research intervention aimed at improving screening coverage of women attending the HIV clinics and, in turn, encouraging those women presenting for screening to check their HIV status. When this study, supported by a Royal Society of Tropical Medicine and Hygiene (RSTMH) small grant^8^ commenced in August 2019, only 31.3% eligible women utilizing the HIV clinic had accessed cervical screening (55). One of the advantages of integrating the clinics arose from the fact that HIV positive women are required to visit the HIV care clinics (1–6 monthly) for reviews and drug refills. This creates a valuable ‘window’ to encourage women to access cervical screening. The evaluation of this cycle involved qualitative interviews with 16 of the health professionals engaged in the screening process at Kagote Health Centre to gauge their perceptions and experiences of service integration. The interventions (based on the findings of the interviews) consisted of sensitizing women about cervical cancer prevention and creating a system of ‘call and recall’ of women due for screening. The latter involved use of an appointment book and cervical screening cards, similar to the cards being used in HIV care to monitor patients’ viral loads. The cards were attached to each of the women’s records to prompt clinicians to discuss and update women on their screening appointments. VHTs and other health professionals in the HIV clinic were mentored to sensitize and refer eligible women for screening.

The intervention to integrate the 2 clinics involved sensitizing HIV positive women about cervical screening and other women attending screening about the need for HIV testing (a bi-lateral process). The word ‘cancer’ is avoided at this stage as the association of cancer with major costs and mortality often means that women in Uganda would prefer not to know. A cancer diagnosis in an LMIC setting such as Uganda where treatment options are very limited can cause severe anxiety and distress. To avoid overwhelming women on first diagnosis of HIV sensitisation commenced after their second visit. Evaluation of this action-research cycle included analysis of patient data and interviews with health workers to assess their perspective of service integration and the impact of this on their workload and on patient outcomes.

All the health worker respondents spoke positively about the integration of cervical screening services with the HIV clinic. The midwife below highlights how it would support follow-up, minimize transport costs and increase access for cervical screening services:

It is good because when it’s an ART clinic day, you get more clients - this is a gathering centre where they come to pick their treatment, it is very good for this client to have all services at once and she goes back rather than giving her a return date. In Africa, people have many challenges on transportation, so she may not come back if you give a different date. But if you put services together, both ART and cervical screening, it reduces on the transport costs for the client (HCP 10 – midwife).

Another respondent acknowledges the value of using this ‘gathering’ of women as a health education ‘moment’ and suggests that women respond very well to this:

The turn up of the women after the health talks in the ART and the immunization clinics is good. The women are responsive (HCP 3 – nurse).

Integrating services is time saving, as the women can access all the required services from one facility instead of looking for services that are scattered in different locations or even visiting the same facility on different days for different services offered. Another perceived benefit of integration is the provision of a better means of record keeping – having all the patient’s details in one facility. This helps clinicians to have a better picture of each of the patients when presenting with any concerns:

If it’s connected to the ART clinic, anyway, then everything is in one case and you even have it in one folder you can treat the person as one (HCP 1 – midwife).

One of the most frequent perceived benefits by almost all the participants is the improved targeting of the women at high-risk for cervical cancer:

It’s a good thing to make cervical screening a routine in ART because their immunity is suppressed; their bodies have some cells that don’t function as well as in those without the virus. So, they have a higher chance of getting the cancer. It is therefore good specially to tell them to be screened every year (HCP 4 – VHT).

The service integration approach proved highly successful yielding an exponential increase in timely screening of HIV positive women: from 31% to nearly 80% (recommended target for any screening programme) over a period of 10 ten months (55).

As with any implementation research the context does not stand still and wait for tidy results. In the last 2 years, the Ugandan Ministry of Health, through international ‘implementing’ partners (including US-financed NGO (Baylor), PEPFAR and Mildmay) introduced a scheme to target HIV positive women. This was later supported by a (short-lived) Results-Based-Finance (RBF) scheme providing financial incentives to health facilities for screening of HIV positive women. K4C has serious concerns about RBF and its impact on service planning and health worker behavior. In practice RBF tends to stimulate a very narrow focus on the outcomes for which remuneration is available often to the exclusion of other needs or patients. At the present time in Uganda cervical cancer screening is driven by *pro rata* payments for women who are HIV positive leading to the almost total neglect of other women.

Table 3 presents the totals for K4C-supported screening and treat services across all facilities. Since 2019 a total of 3,690 women have accessed cervical screening. Of these an average of 6% had positive results and treatment. Seventy-four percent of those screened were HIV positive. Table 3 also enables us to look at screening profiles by health facility and gives some indication of the impact of K4Cs health awareness intervention, predominantly in Kagote; at least in terms of encouraging those women who are not HIV positive (and therefore not already connected with health facilities) to attend screening.

**Table 3:** Number of women screened by HIV status and screening outcomes by health facility (April 2019 to date).

	Overall % (*n*)	Kagote % (*n*)	Bukuuku % (*n*)	Kasusu % (*n*)	Kira % (*n*)	Kasangati % (*n*)
Total *n*	3,690	1,499	1,088	163	294	173
HIV + ve	74 (2728.6)	52.9 (793.5)	83.1 (904)	84 (136.9)	91.2 (267.6)	92.5 (160)
Ca + ve	6 (221.4)	5.6 (83.9)	16.7 (181.7)	1.8 (2.9)	3.1 (9.1)	4 (6.9)

The outreach health education program described above has only taken place in Kagote. While Auma’s work to integrate HIV and screening services has also only taken place at Kagote the outcome data suggest that the RBF intervention has overwhelmed approaches to screening in the past 2 years. A serious unintended consequence of this has been the almost total neglect of women who are not HIV positive (over 90% of women screened in some facilities are HIV positive compared to a rate of 52.9% in Kagote). This provides a very powerful critique of RBF-driven interventions. As is common with interventions driven by external (foreign) partners, this time-limited nature of financial support will inevitably simply put a halt to any screening in future. K4C is currently trying to rebalance this with a focus on awareness-raising post-natal and immunization clinics. In some facilities where the screening takes place within the HIV clinics, we are concerned both that staff will fail to screen HIV negative women but also such women may want to avoid the stigma of visiting the HIV clinics. In one health facility where this has emerged as a key concern, K4C has established a new screening clinic adjacent to maternity open to any women.

## 4. Conclusion

This paper reports on the evolution of a cervical screening program that has developed and become embedded in public health services in one health district in Uganda over the past 5 years. As we noted, earlier effective and sustainable implementation research is a continuous process typically involving a range of evidence-based interventions informed by consecutive ‘cycles’. The decision to report, at this juncture, was informed by the launch of a new EVA device supported by Artificial Intelligence. The telemedicine aspect of the EVA system, where expert clinical advice and support with clinical decision making supported through virtual image sharing and consultation in real time, has proved effective in preventing diagnostic drift and ensuring women receive appropriate treatment. Respectful care lies at the heart of all services and plays a major role in promoting access, particularly in such sensitive areas as cervical cancer and HIV. A foundation of respectful care coupled with comprehensively trained multi-disciplinary teams and strategically planned public awareness programs (guided by GIS) has been shown capable of delivering high quality point-of-care preventive services. The integration of cervical screening with HIV clinics took this to another level ensuring that those women most at risk access these services. Service integration is also an important component of our sustainability commitment. K4C’s commitment to co-working in public services has ensured a model that can be sustained with minimal external resourcing beyond the management of the EVA device. The effectiveness of the thermocoagulation approach has been picked up by the Ministry of health who now provide these devices. The updated next-generation EVA device enhances this telemedicine support through an Artificial Intelligence (AI) automated clinical decision support tool embedded within the system. AI presents unique opportunities to extend the task-shifted model and increase the efficacy, availability and quality of screening. The next phase of the work will involve the planned introduction of the AI approach in 2 carefully monitored sites to evaluate the contribution that this can make to cervical screening in Uganda. We also plan to assess the potential for frugal innovation returns to the UK’s National Health Service through this innovation.

## Data availability statement

The original contributions presented in the study are included in the article/supplementary material, further inquiries can be directed to the corresponding author.

## Ethics statement

Ethical approval was obtained from the University of Salford. Written informed consent was obtained from the individuals involved, for the publication of any potentially identifiable images or data included in this article.

## Author contributions

LA, JAJ, JA, and AN conceived the project. LA, JA, and AN wrote the manuscript. MS supported data analysis. VK led the training. All authors reviewed drafts and approved the final manuscript.

## Conflict of interest

The authors declare that the research was conducted in the absence of any commercial or financial relationships that could be construed as a potential conflict of interest.

## Publisher’s note

All claims expressed in this article are solely those of the authors and do not necessarily represent those of their affiliated organizations, or those of the publisher, the editors and the reviewers. Any product that may be evaluated in this article, or claim that may be made by its manufacturer, is not guaranteed or endorsed by the publisher.

## Footnotes

^1^www.knowledge4change.org

^2^Testing for the presence of HPV is now commonly used as a triage process reducing the need for more invasive PAP smear testing (18, 19).

^3^Further information and guidance on the practical use of both thermocoagulation and the EVA device is available on the Mobile ODT website 9 with free demonstrations available: https://www.mobileodt.com/products/eva-pro/.

^4^The 3-month fellowships coincided with the start of the COVID-19 pandemic so in practice this training also took place on a virtual basis.

^5^K4C has a portfolio of resources that is regularly updated and would be happy to share.

^6^Funded by the UK Department for International Development [now the UK Foreign, Commonwealth & Development Office (FCDO)].

^7^Ethical Approval for this work was obtained HSR1819-061 (for interviews with health workers) and HSR1819-061 (for the GIS work).

^8^Funded by the National Institute for Health and Care Research (NIHR).

## Abbreviations

AI: Artificial Intelligence
EVA: Enhanced Visual Assessment
GIS: Geographic Information Systems
HIV: Human Immunodeficiency Virus
HPV: Human Papillomavirus
LMIC: Low- and Middle-Income Countries
NGO: Non-Governmental Organizations
PAP: Smear Papanicolaou Smear test
RBF: Results Based Finance
VHT: Village Health Team
VIA: Visual Inspection with Acetic Acid
